# The coSIR model predicts effective strategies to limit the spread of SARS-CoV-2 variants with low severity and high transmissibility

**DOI:** 10.1007/s11071-021-06705-8

**Published:** 2021-07-23

**Authors:** Longchen Xu, Haohang Zhang, Hengyi Xu, Han Yang, Lei Zhang, Wei Zhang, Fei Gu, Xun Lan

**Affiliations:** 1 School of Life Sciences, Tsinghua University, Beijing, 100084 China; 2 Tsinghua-Peking Joint Center for Life Sciences, Tsinghua University, Beijing, 100084 China; 3 Eight-Year MD Program, Peking Union Medical College, Beijing, 100084 China; 4 Department of Basic Medical Science, School of Medicine, Tsinghua University, Beijing, 100084 China; 5 MOE Key Laboratory of Bioinformatics, Tsinghua University, Beijing, 100084 China; 6 DAMO, Alibaba Cloud Intelligence Business Group, Alibaba Group, Hangzhou, 310052 China

**Keywords:** COVID-19, Competitive evolution, SIR model, SARS-CoV-2 evolution, COVID-19 control strategy

## Abstract

Multiple new variants of SARS-CoV-2 have been identified as the COVID-19 pandemic spreads across the globe. However, most epidemic models view the virus as static and unchanging and thus fail to address the consequences of the potential evolution of the virus. Here, we built a competitive susceptible-infected-removed (coSIR) model to simulate the competition between virus strains of differing severities or transmissibility under various virus control policies. The coSIR model predicts that although the virus is extremely unlikely to evolve into a “super virus” that causes an increased fatality rate, virus variants with less severe symptoms can lead to potential new outbreaks and can cost more lives over time. The present model also demonstrates that the protocols restricting the transmission of the virus, such as wearing masks and social distancing, are the most effective strategy in reducing total mortality. A combination of adequate testing and strict quarantine is a powerful alternative to policies such as mandatory stay-at-home orders, which may have an enormous negative impact on the economy. In addition, building Mobile Cabin Hospitals can be effective and efficient in reducing the mortality rate of highly infectious virus strains.

**Supplementary Information:**

The online version contains supplementary material available at 10.1007/s11071-021-06705-8.

## Introduction

Severe acute respiratory syndrome coronavirus 2 (SARS-CoV-2) remains one of the top public health concerns worldwide 1 year after its identification [1–3]. There have been signs of stabilization in key countries since last August; however, since October 2020, the global COVID-19 epidemic appears to be on the rebound. The second outbreak exceeds the first wave in many aspects, and it raises the concern that COVID-19 may eventually lead to a yearly cyclic “lethal flu” until effective vaccines are widely administered.

Although SARS-CoV-2 has a relatively low genomic diversity [4] due to its proofreading mechanisms [5], natural selection can accelerate the rapid spread of environmentally favourable variants. Since the outbreak of the epidemic, novel SARS-CoV-2 strains have been described in many studies and reports [6–13]. While new strains with higher transmissibility emerged frequently—the D614G mutant replaced the original virus strain as the majority in just four months [6], and the N501Y mutant was approximately 70% more infectious [13]—the lethality of most of the new virus strains tended to remain the same or decreased, which had been suggested by several previous researches [6–8, 14, 15].

Individuals infected by one strain of the virus are likely to acquire immunity that is cross-protective against other strains [16, 17]; thus, competition among strains is one of the major driving forces of the evolution of the virus. Under distinct pandemic response strategies, the competitive dynamics of the virus strains, with varying degrees of transmissibility and severity, can be significantly different. With no end of the pandemic in sight and the frequency of new strains emerging, the study of the evolutionary trajectory of SARS-CoV-2 is crucial for planning future virus prevention strategies. Appropriate and timely actions may help to manage the competition of SARS-CoV-2 strains and to facilitate their evolution towards lower pathogenicity.

Various mathematical models have been developed to simulate the dynamics of COVID-19, and a majority of them are based on the classic Susceptible-Infected-Removed (SIR) model [18–23]. For example, Brett and Rohani [18] evaluated the herd immunity strategy by transmission analysis; Weitz et al*.* [20] simulated the pandemic progress based on shield immunity; and Wilder et al*.* [21] constructed a modified SIR model considering population variation. These models predicted the trajectory of the pandemic without considering the competition among different SARS-CoV-2 strains. Competitive models also exist. For example, Naji and Hussien [24] have simulated virus competition by a “cross-immune score”; Poletto et al*.* [25] attempted virus competition modelling in the context of spatial distribution. However, these models were built on previous pandemics and thus cannot properly reflect the infectious ability of SARS-CoV-2 at the asymptomatic stage.

Here, we developed a coSIR (competitive SIR) model to study the impact of pandemic management policies on competition among various SARS-CoV-2 strains. The coSIR model showed that the virus is likely to evolve to be more infectious and less symptomatic, giving it the ability to infect more individuals without being easily detected and isolated. It also predicted that virus transmission control policies, such as social distancing and mask-wearing, are most effective in dealing with the low-severity and highly infectious virus variants. Combining adequate nucleic acid testing (NAT) and quarantine can also achieve a desirable effect in controlling the spread of the virus. The simulations also demonstrated that building mobile cabin hospitals is an effective and efficient strategy to reduce the mortality rate of highly infectious virus variants.

## Results

### The coSIR model

**Fig. 1:**
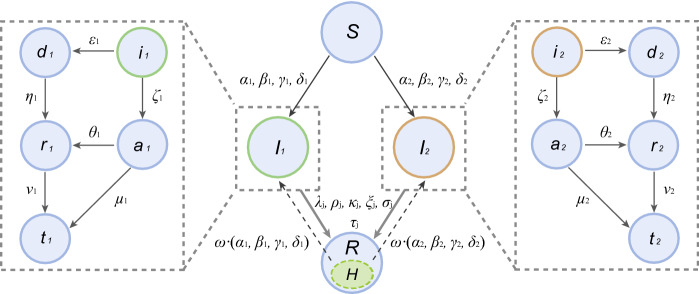
Simplified schematic of the coSIR model. Graphic representation of the interactions between the different stages of infection. *S*, susceptible; *I*_*j*_(*j*=1 2), infected of the *j*th virus strain; *R*, removed. The infected population can be further divided into 5 subgroups:* i*_*j*_ (asymptomatic infected, undetected); *d*_*j*_, diagnosed (asymptomatic infected, detected); *a*_*j*_, ailing (symptomatic infected, undetected); *r*_*j*_, recognized (symptomatic infected, detected); *t*_*j*_, threatened (infected with life-threatening symptoms, detected). Arrows from *S* to *I*_*j*_ only enter *i*_*j*_, while arrows from *I*_*j*_ to *R* are from all 5 subgroups of *I*_*j*_. Stage *R* (removed) consists of *H* (healed) and *E* (extinct), and the dashed arrows from *H* to *I*_*j*_ represent the small chance of healed individuals becoming reinfected. Each Greek letter represents the transition probability from one stage to another. For example, $\alpha _{j} ,\beta _{j} ,\gamma _{j} ,\delta _{j}$ represent the probabilities a susceptible individual was infected after contacting with $i_{j} ,d_{j} ,a_{j} ,r_{j}$, respectively. The transition probabilities from 5 subgroups of *I*_*j*_ to *H* are $\lambda _{j} ,\rho _{j} ,\kappa _{j} ,\xi _{j} ,\sigma _{j}$, while only *t*_*j*_ (the threatened) can transit directly into *E* with probability $\tau _{j}$

The coSIR model, consisting of an SIR dynamics component and a strain competition component, can be used to simulate the competitive relationship between two coexisting virus strains (Fig. 1, Table 1). The SIR dynamics component is mainly adopted from the SIDARTHE model [19].

**Table 1:** Nomenclature

Symbol	Meaning
*S*	Susceptible
*I*_*j*_	Infected of the *j*th virus strain
*R*	Removed
*H*	Healed
*E*	Extinct
OI	Overall infections
OM	Overall mortality
*i*_*j*_	Undetected asymptomatic patients
*d*_*j*_	Diagnosed (detected asymptomatic patients)
*a*_*j*_	Ailing (undetected symptomatic patients)
*r*_*j*_	Recognized (detected symptomatic patients)
*t*_*j*_	Threatened (infected with life-threatening symptoms, detected patients)
$\alpha _{j} ,\beta _{j} ,\gamma _{j} ,\delta _{j}$	Infection rate after contacting with *t*_*j*_, *d*_*j*_, *a*_*j*_,*r*_*j*_ respectively
$\varepsilon _{j} ,\theta _{j}$	Diagnosis rates of patients with state *i*_*j*_, *a*_*j*_ respectively
$\zeta _{j} ,\eta _{j}$	Symptomatic rates of patients with state *i*_*j*_, *d*_*j*_ respectively
$\mu _{j} ,v_{j}$	Occurrence rate of life-threatening symptoms in *a*_*j*_, *r*_*j*_ respectively
$\tau _{j}$	Mortality rate of threatened patients
$\lambda _{j} ,\kappa _{j} ,\xi _{j} ,\rho _{j} ,\sigma _{j}$	Cure rate of patients with state *t*_*j*_, *d*_*j*_, *a*_*j*_, *r*_*j*_, *t*_*j*_, respectively
$\omega$	Risk reduction rate of reinfection for individuals who have been healed
*C*_infection_	Score used to value the difference of infections between two strains
*C*_mortality_	Score used to value the difference of mortality between two strains

As shown in Fig. 1, the population was partitioned into 3 groups: *S*, susceptible; *I*_*j*_(*j* = 1, 2), infected with the *j*th virus strain; and *R*, removed (consists of *H* (healed) and *E* (extinct)). The infected individuals (*I*_*j*_) can be further divided into 5 subgroups:* i*_*j*_ (asymptomatic infected, undetected); *d*_*j*_, diagnosed (asymptomatic infected, detected); *a*_*j*_, ailing (symptomatic infected, undetected); *r*_*j*_, recognized (symptomatic infected, detected); and *t*_*j*_, threatened (infected with life-threatening symptoms, detected). In this model, the 2 different virus strains (*j* = 1, 2) compete for the same susceptible population. A few reports have also suggested that healed individuals have a small chance of being reinfected [26], and cross-infection may also occur with coexisting SARS-CoV-2 subtypes [16, 17]. To reflect this, the healed individuals were subjected to a small chance of being reinfected (arrows from *H* to *I*_*j*_). Each Greek letter represents the transition probability from one stage to another. For example, $\alpha _{j} ,\beta _{j} ,\gamma _{j} ,\delta _{j}$ represent the probabilities a susceptible individual was infected after contacting with $i_{j} ,d_{j} ,a_{j} ,r_{j}$, respectively. The transition probabilities from the 5 subgroups of *I*_*j*_ to *H* are $\lambda _{j} ,\rho _{j} ,\kappa _{j} ,\xi _{j} ,\sigma _{j}$, while only *t*_*j*_ (the threatened) can transition directly into *E* (extinct) with probability $\tau _{j}$. Detailed specifications are described in the Methods section.

The impact of the virus on an individual can be extremely complex; for simplicity, we quantified the overall impact of a virus strain as the total number of infections or mortality throughout the pandemic. Two main characteristics of the virus are considered, namely severity and transmissibility. The evolutionary trajectory of these two properties of the virus was modelled separately, as one mutation changing both properties is rare.

Four levels of controlling strength were considered to represent a variety of environments across the world: (1). Free Development—almost no virus management strategies implemented; (2). Weak Control—increased self-isolation; (3). Medium Control—increased self-isolation and social distancing; (4). Strict Control—increased self-isolation, social distancing, and a broad diagnosis campaign (for detailed parameter specifications, see Methods and Table S1). Under the specifications of the four levels of virus control, we simulated 4 common policies that are reported to be highly effective [19, 27–30]: (1). Virus transmission control (Block in short)—reduces the transmission rate of the virus in a population, including mask-wearing and social distancing; (2). Increased medical investment (Cure in short)—improves the cure rate for both virus strains simultaneously; (3). Isolation of confirmed patients (Isolation in short)—significantly reduces the transmission rate of the virus from confirmed patients; and (4). Extensive testing of the population (Screen in short)—increases the diagnosis rate of infected individuals regardless of their symptoms (for model parameters affected by the 4 policies see Methods, Table S2).

The simulation is robust under different initial infection ratios of the original strain to the emerging strain, and we set the ratio at 10,000:100 for simplicity (Methods, Fig. S1). In the following sections, we used log ratio of the overall infections of the emerging strain during the entire pandemic to that of the original strain as a measure to describe relative competitiveness of the emerging strain over the original strain. The total population size was set to 60,000,000, which is the same as in the SIDARTHE model [19].

### Virus strains with low symptom severity are likely to spread more quickly

A question constantly attracts the public is whether SARS-CoV-2 could evolve into a “supervirus” and become even deadlier than it already is. In this section, the coSIR model is utilized to simulate the competitive dynamics when a new virus strain with different severities appears. The impact of symptom severity on the competitiveness of strains is complicated. On the one hand, increased severity makes it easier for infected individuals to be diagnosed and isolated; on the other hand, high-severity symptoms may take a longer time to resolve, allowing more time for the virus strain to spread. We simulated the competition among strains of different symptom severities by changing the relative severity of the emerging strain while setting the severity of the original strain as a reference.

The present model predicted that when the severity of the emerging strain is higher than that of the original strain, the original strain always prevails. The competitiveness of the emerging strain is inversely proportional to its symptom severity. The lower the severity of the emerging strain is, the higher the probability it wins. Although differences exist in the competition dynamics under various policies and control levels, the overall trend is robust (Fig. S2). In all cases simulated, only an emerging strain with low symptom severity may outcompete the original strain, suggesting that SARS-CoV-2 is more likely to evolve towards lower symptom severity.

**Fig. 2:**
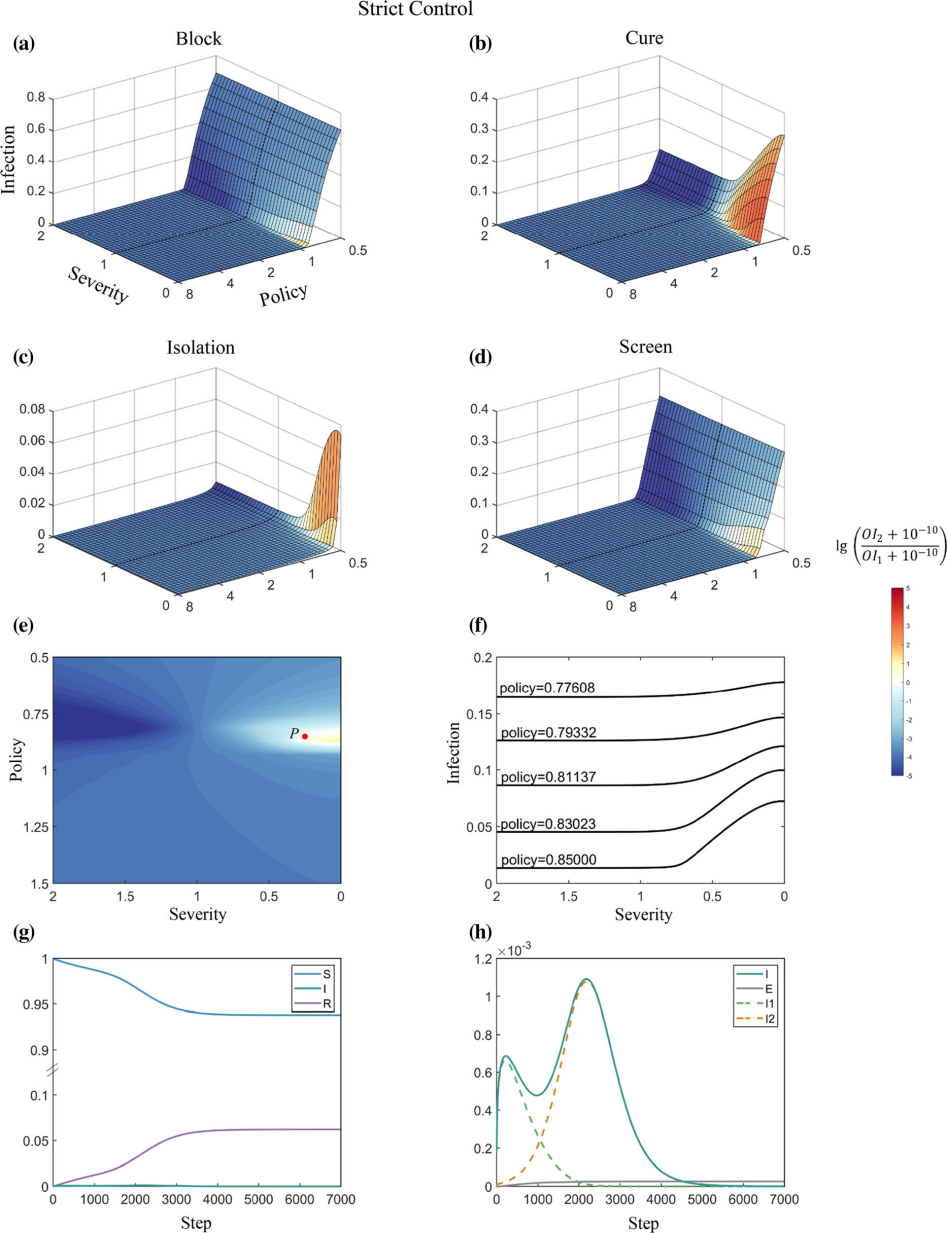
Competition between virus strains with different severities under Strict Control. For **a**–**d**, the x-axis represents the severity of the emerging strain relative to that of the original strain, which is set to 1. A value < 1 means that the severity of the emerging strain is lower than that of the original strain and vice versa. The y-axis represents policy strengths, which is the factor used to multiply (or divide) the specific parameters related to the four respective policies in the coSIR model (for policy-related parameters, see Methods and Table S2). The z-axis represents the overall infection rate among the whole population. The colour bar represents the log ratio of the number of overall infections (OI) of the emerging strain to that of the original strain. A positive number means that the emerging strain outcompetes the original strain in terms of the number of infections. **e**, the 2-dimensional top view of **a**, showing that a milder emerging strain can surpass the original strain in the number of infections under suitable conditions. **f**, impact of severity on the overall infection rate under various strengths of the Block policy. **g** and **h,** details of the epidemic dynamics for the two competing strains of point *P* in **a**

**Fig. 3:**
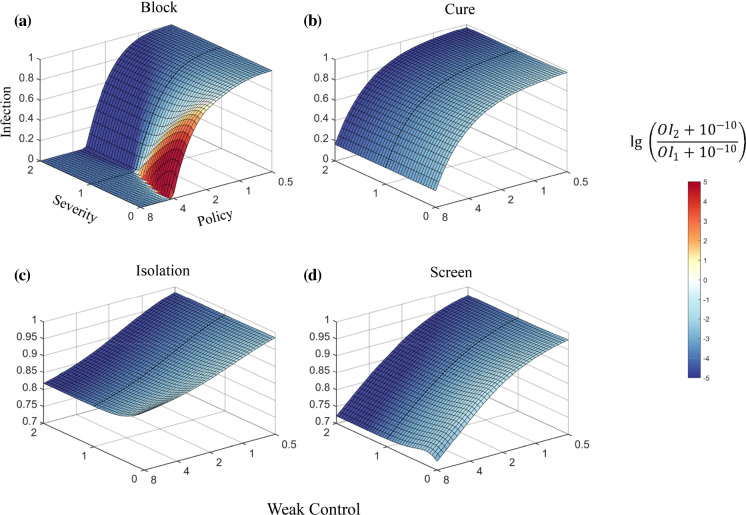
Competition between virus strains with different severities under Weak Control

Stringent external policies exert a strong negative selective pressure on more severe strains, which are more likely to be detected. The more stringent the policy is, the higher the probability that a strain with a lower severity will eventually prevail (Figs. 2, 3). However, under an extremely stringent policy, the spread of both virus strains is greatly suppressed, and the evolution of the virus is slowed. Under an extremely relaxed policy, both strains can spread freely until almost the whole population is infected.

Under Strict Control, the difference in policy strength has a greater impact on the competition results (Fig. 2). However, under Medium or Weak Control, the impact of policy strength gradually decreased (Figs. 3, S2). For instance, the simulation of Isolation and Screen under Weak Control reveals an approximately “parallel line” pattern, indicating that the competition dynamics are dominated by the severity of the emerging strain, while policy strength has a negligible effect. These results suggest that the effect of a policy is often affected by the overall strength of virus control. Under a loose controlling background, the impact of the policy on competition dynamics is greatly suppressed. Cure policy shows a stronger impact on the overall infection rate compared to Isolation and Screen under Weak Control (Fig. 3b–d). Block policy reveals a significant influence on restricting the spread of the virus and on the competition dynamics between the virus strains, indicating its critical role in pandemic prevention under an overall weak control environment (Fig. 3a).

### Virus strains with lower symptom severity may lead to more deaths

A virus strain with a lower symptom severity can gain competitive advantages over the original strain; however, the overall impact of the emerging strain on the mortality of the population throughout the pandemic is not clear. Figures 2, 3 suggest that a high overall infection rate in the population is always linked with the rapid onset of a low-severity emerging strain. With a lower severity in the emerging strain, the impact of low-severity strain increases, and there will be a larger total number of infections. This trend is illustrated in Fig. 2e, which is a section diagram of Fig. 2a with policy strength being set at different levels.

When the severity of the emerging strain is higher (ratio > 1), the total number of individuals infected by the emerging strain is negligible. When the severity is lower (ratio < 1), the total number increases as the severity decreases. However, the total mortality is not always positively correlated with the number of overall infections since a reduced severity of the emerging strain also affects the diagnosis rate and the case-fatality ratio. Total mortality is a complex product of two negatively correlated variables, namely the number of infections and case-fatality ratio. In the following simulations, we focused on the number of deaths caused by the two competing virus strains instead of the number of infections.

**Fig. 4:**
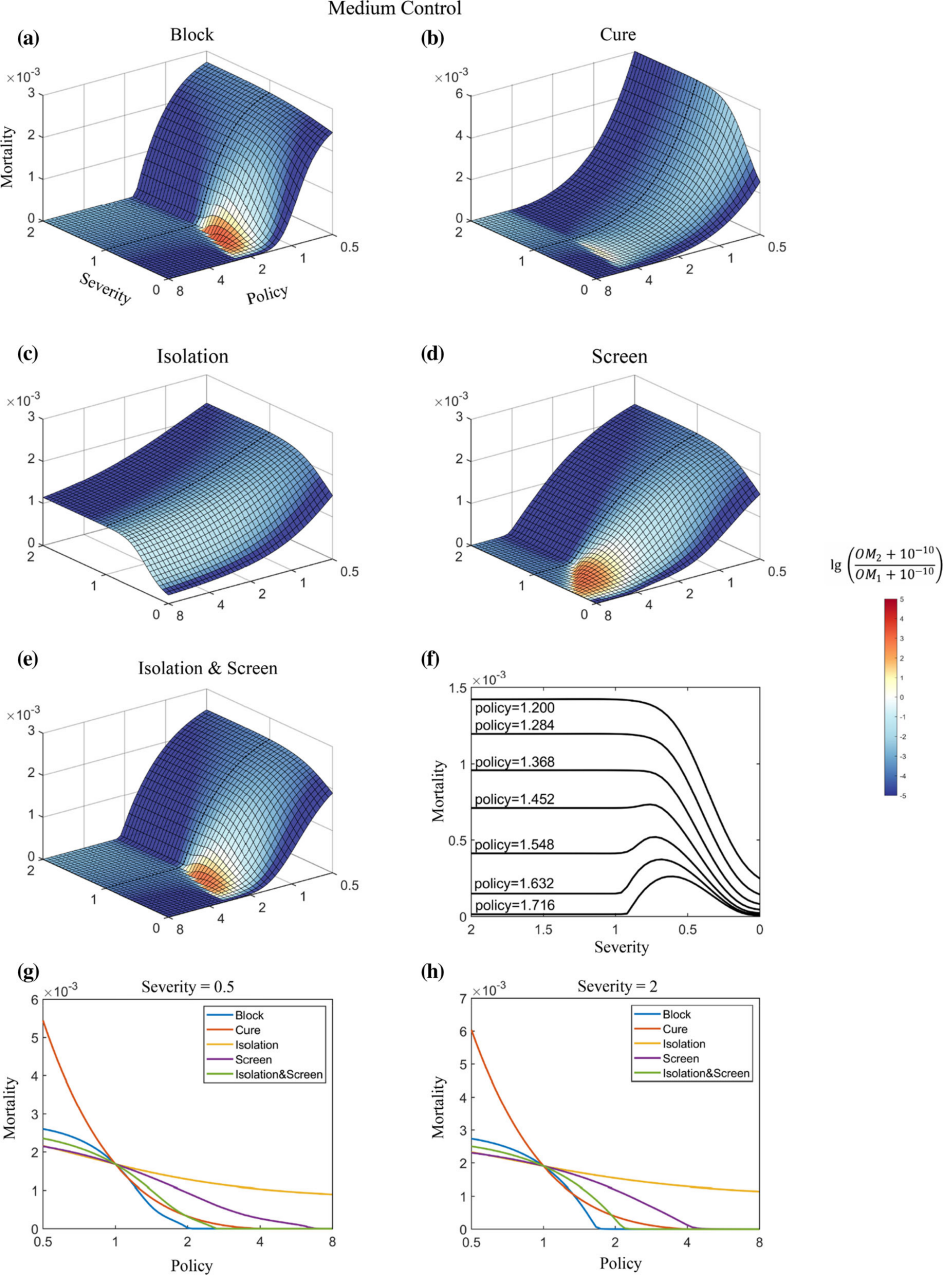
Mortality prediction for two competing strains with differing severity under Medium Control. For **a**–**d,** the x-axis represents the severity of the emerging strain relative to that of the original strain, the y-axis represents policy strengths respectively, and the z-axis represents the total number of deaths. The colour bar represents the log ratio of the overall mortality (OM) caused by the emerging strain to that caused by the original strain. A positive number means that the emerging strain outcompetes the original strain. **e**, the combined effect of Isolation and Screen policies. **f**, the left cross-sectional view of **a**, and each curve represents the mortality as a function of strain severity under a specific Block level. **g** and **h,** mortality under different policies when the emerging strain was at the indicated severities relative to the original strain

In the simulations, two policies—Block and Screen—share the following patterns regarding the total number of deaths (Fig. 4a, d). (1). Under strong policies, viral competition has little effect on the final death rate. In this case, both virus strains are suppressed by intensive policies, and the death toll remains relatively low. (2). Under relaxed policies, both viruses can spread in an approximately unimpeded way in a population. A positive correlation could be observed between the mortality rate and the severity of the emerging strain. The milder the emerging strain is, the fewer the total number of deaths. (3). Under intermediate policies, the total number of deaths rises first and then falls as the severity of the emerging strain declines. This can be explained by the shifted balance between the opposing effects of the decreased severity. As the severity of the emerging strain decreases, the total infection number increases as indicated in the previous section, while the case-fatality ratio decreases. When virus severity is only slightly reduced, the increased number of infections has a stronger impact on the number of deaths than the reduced case-fatality ratio does, resulting in a slight increase in the total mortality. However, when the virus severity was greatly reduced, the effect of a decreased case-fatality ratio will dominate the calculation of total mortality.

Unlike the other three policies, under the Cure policy, the mortality of the emerging strain rarely surpasses that of the original strain, and the overall mortality rate is always proportional to the severity of a milder emerging strain, suggesting that Cure can be an effective policy in dealing with the increased mortality caused by low-severity strains. Notably, a decrease in the Cure policy would markedly increase mortality (Fig. 4g, h). Increased hospitalizations due to a growing number of total infections may overwhelm the local health care system and lead to a sharp decline in the Cure policy. Therefore, to avoid a high mortality rate, it is crucial to control the number of infections to maintain the Cure policy at an adequate level.

Block policy has the strongest virus control power among the four policies. A strengthened Block policy can most efficiently suppress the spread of the virus regardless of the severity of the emerging strain. (Fig. 4a, g, h). Compared with the other policies, the Isolation policy shows a relatively low effect on the total mortality even when it is rigorously enforced because the policy affects only confirmed patients. However, a combination of Isolation and Screen policy can have approximately the same effect as the Block policy. This suggests that adequate testing combined with quarantine can serve as an effective alternative to policies such as mandatory stay-at-home orders.

### Mobile cabin hospitals are effective and efficient in controlling highly infectious strains

**Fig. 5:**
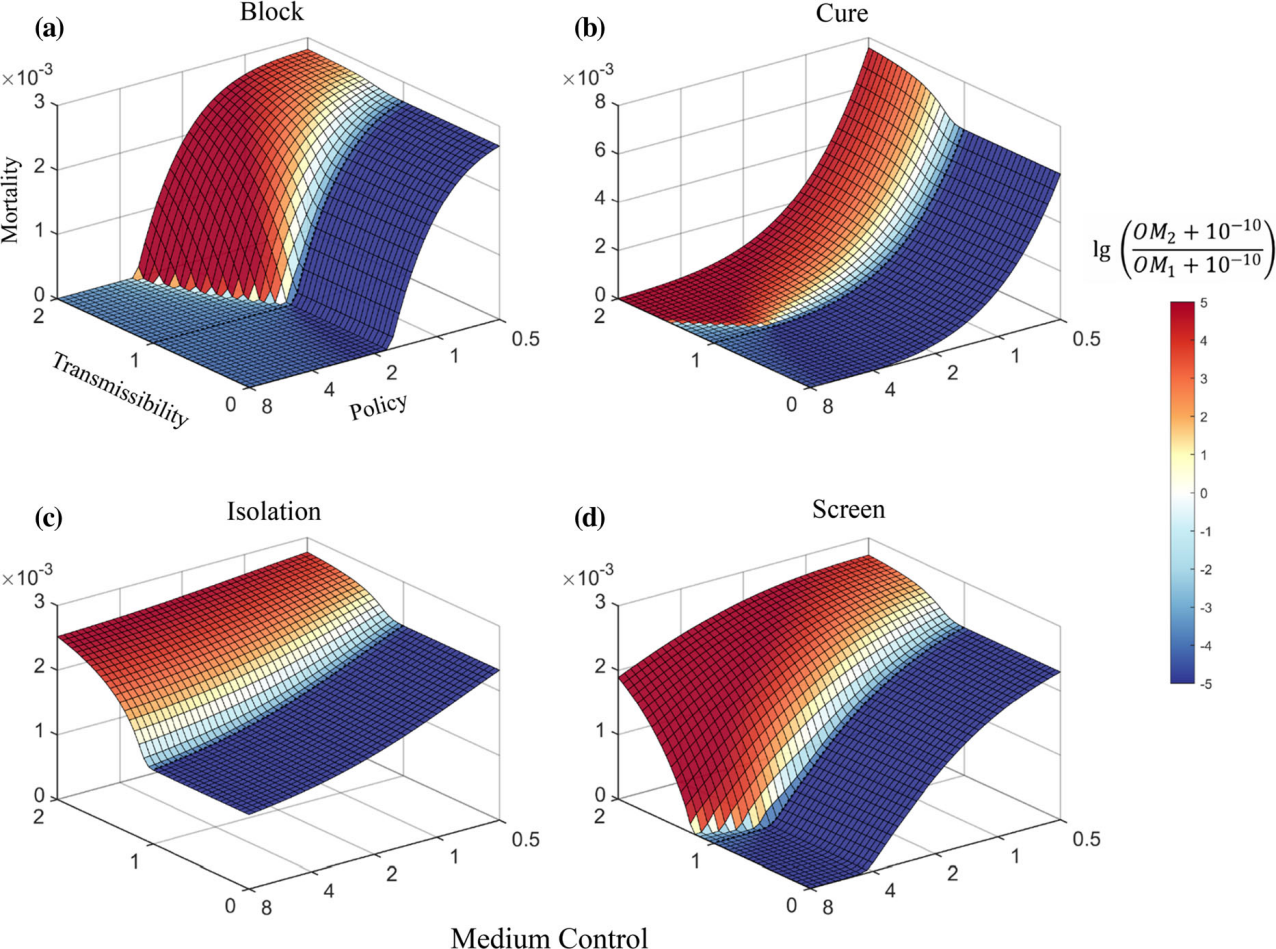
Mortality prediction for two competing strains with differing transmissibility under different policies and Medium Control

Next, we simulated the competition dynamics of the strains with differing transmissibility to study the effects of different policies on the spread of highly infectious strains. As expected, the change in transmissibility has a more significant and straightforward influence on the competition dynamics. The emerging virus strain with a small enhancement in transmissibility can outcompete the original strain in most cases. The total number of infections and deaths of the emerging strain was positively correlated with its transmissibility (Figs. 5, S3).

**Fig. 6:**
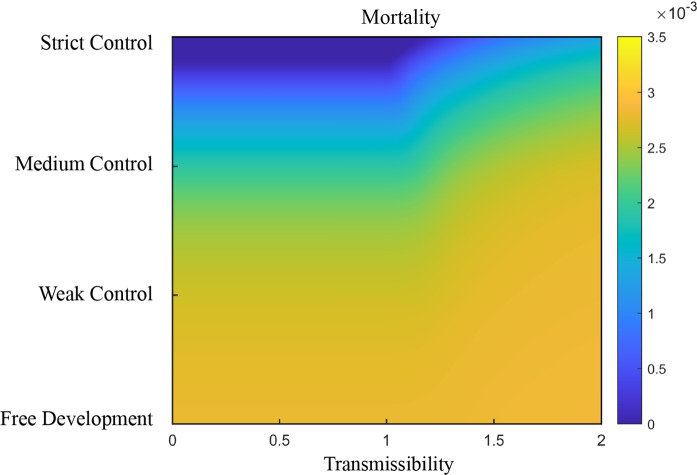
Mortality prediction for two competing strains with differing transmissibility under different controlling levels. Mortality decreases as the level of control increases from Free Development to Strict Control under default parameters (Methods and Table S1)

**Fig. 7:**
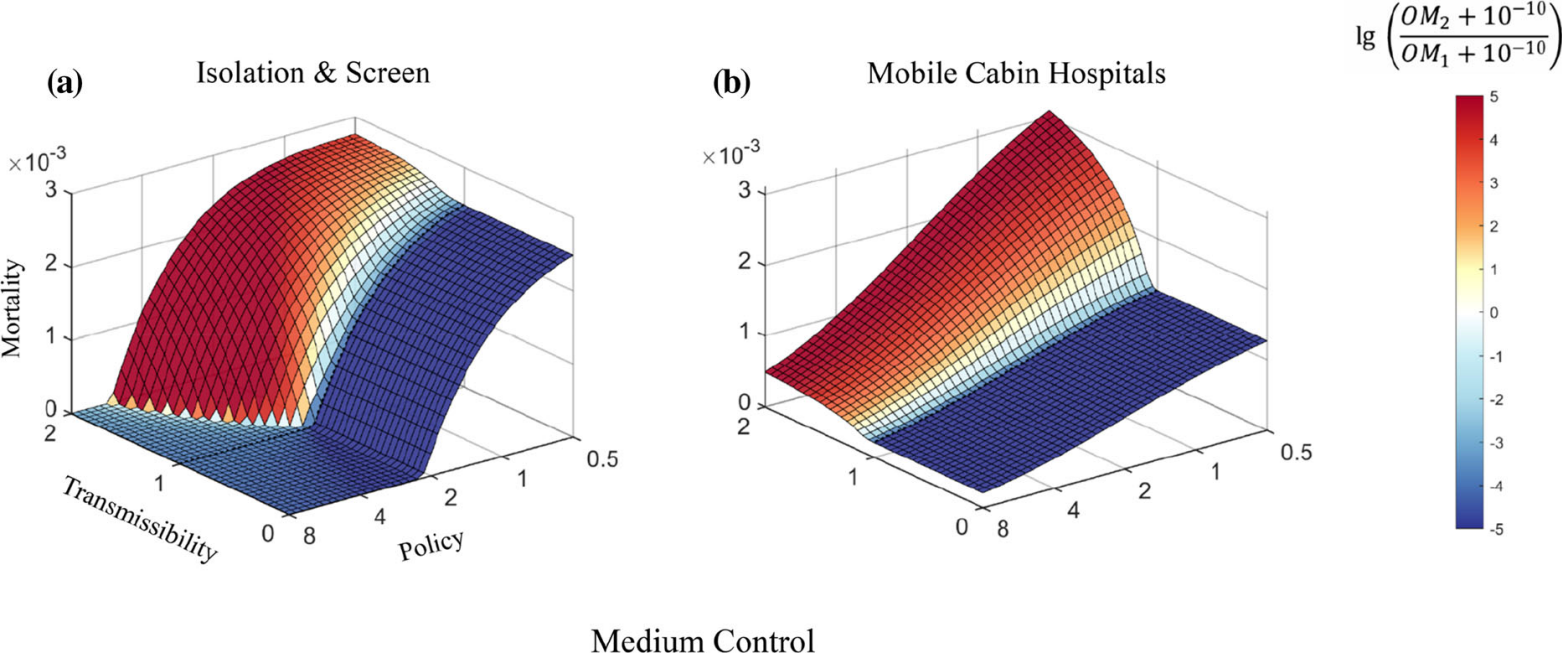
Mortality prediction under mixed policy and Medium Control. The influence of Mobile Cabin Hospitals on mortality shows that they are an effective policy for controlling highly transmissible virus strains

As a result, the higher the transmissibility of the emerging strain, the more stringent policies are required to limit the number of mortality (Fig. 6). Block policy has a strong ability to control highly infectious strains. The present model shows that the inhibitory effect of policies is weakened in the presence of highly contagious strains. However, after combining a high level of control and a stringent Block policy, the final death toll can be kept at a lower level (Fig. 5a). Although a high cure rate can significantly reduce the total number of deaths caused by a highly infectious strain, improving the effectiveness of COVID-19 therapies may require major advances in our understanding of the virus, which requires time. In contrast, Isolation and Screen policies have very limited effects on controlling the overall mortality rate on their own. However, a mixed strategy of these two policies can achieve a similar effect as the Block policy (Figs. 5, 7a).

In addition to more stringent control, Mobile Cabin Hospitals, a measure demonstrated to be highly effective in past outbreak practices [31–33], showed high potential in the control of mortality in our simulation (Fig. 7b). By isolating infected individuals confirmed with mild symptoms, Mobile Cabin Hospitals can block the transmission of the virus from diagnosed individuals to the susceptible population [32] (equivalent to Isolation policy) and provide medical support to alleviate life-threatening symptoms [32, 33] (symptom severity control), thus significantly decreasing the death rate regardless of the transmissibility of the virus. The simulations suggest that even with limited resources, building Mobile Cabin Hospitals is an efficient and effective strategy to control highly transmissible strains.

## Discussion

Multiple new variants of SARS-CoV-2, such as the D614G and N501Y mutants [6, 7, 13], were identified in a rather short period. By considering the competition between two different SARS-CoV-2 strains, the present model can provide us with more insight into the epidemic dynamics and evolution of the virus. We note that the coSIR model does not seek to fit the real data and, therefore does not aim to precisely explain the epidemic in a specific country or region. By setting different parameters and initial values, our model may represent conditions in different regions and under different epidemic response strategies. Thus, we can draw robust conclusions that are generalizable under various situations based on the common trend in the evolutionary trajectory of the virus.

**Fig. 8:**
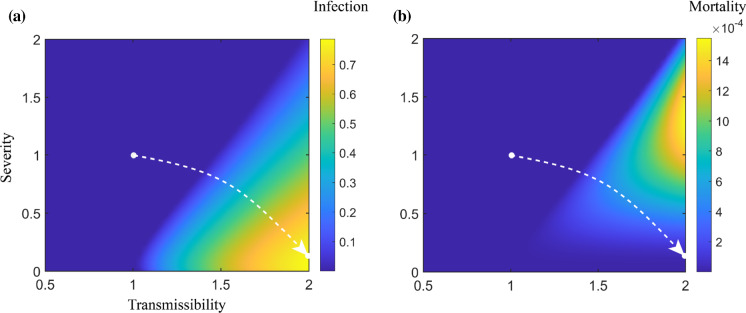
Overall infection and mortality given emerging strains with different transmissibility and severity. When the severity of the emerging strain is kept unchanged, the infection and mortality are positively correlated with transmissibility; given the same transmissibility, the infection will almost always rise as the severity decreases, while the mortality rises first and then decreases. The artificially drawn white dashed line is a possible evolutionary trajectory of the virus towards a higher infection rate (**a**) and a rising and then decreasing mortality rate (**b**)

Compared to the strong influence of transmissibility, the impact of severity of the emerging strain shows a more complex pattern. The coSIR model suggests that the less severe an emerging virus strain is, the more likely it is to outcompete the original strain. More restrictive policies can make it easier for low-severity emerging strains to gain advantages over the original strain by providing greater selection pressure. Under the same severity, the total mortality is positively correlated with the transmissibility of the emerging strain, while given the same transmissibility, the mortality rises first and then decreases as the severity increases (Figs. 8b, S4).

Simulation results of competition between the strains differing in both severity and transmissibility at the same time are shown in Figs. 8a, S4, where the colour indicates the overall infection rate. The present model suggests that the evolutionary trajectory of SARS-CoV-2 is likely towards a more infectious, less severe "flu-like" direction, depicted by the artificially generated white dashed line in the infection contour (Figs. 8a, S4) and the mortality contour (Figs. 8b, S4). Notably, the evolutionary direction of SARS-CoV-2 indicated by the coSIR model can only represent global virus evolution in long-term. In short-term, higher severity strains may emerge locally.

We also derived the result that the total *R*_0_ of the entire competitive system is a weighted average of the two virus strains and is mainly determined by the strain causing more infections. Details are in the Methods section.

For strains that evolve towards a lower severity and a higher transmissibility, the overall numbers of infections in the epidemic will increase, and the mortality rate may rise first and then fall later. Stronger policies, such as mandatory stay-at-home orders, can effectively decrease the rebound of mortality caused by the low-severity strains and bring the epidemic under control. For highly infectious strains, the effects of certain policies such as Isolation and Screen would be greatly hampered, while Block and Cure have more desirable outcomes. Our analysis highlights that Mobile Cabin Hospitals, which reduce both the symptom severity of the disease and the transmission rate among the confirmed population, can be an efficient and effective measure in controlling highly transmissible viruses.

## Methods

The coSIR model consists of two components: the epidemic dynamics component and the strain competition component. The epidemic dynamics component is mainly adopted from the SIDARTHE model [19], with the additional consideration of the possibility of reinfection by introducing a risk reduction rate for healed individuals. The strain competition component simulates the competition between two different virus strains.

### Epidemic dynamics of the coSIR model

The epidemic dynamics of the coSIR model are mainly adopted from the SIDARTHE model, and the ordinary differential equations are as follows:

1 $$  \dot{S}\left( t \right) =  - S\left( t \right)\sum\limits_{{j = 1,2}} {\left( {\alpha _{j} i_{j} \left( t \right) + \beta _{j} d_{j} \left( t \right) + \gamma _{j} a_{j} \left( t \right) + \delta _{j} r_{j} \left( t \right)} \right)} ~ $$

2 $$  \dot{i}_{j} \left( t \right) = \left( {S\left( t \right) + \omega H\left( t \right)} \right)\left( {\alpha _{j} i_{j} \left( t \right) + \beta _{j} d_{j} \left( t \right) + \gamma _{j} a_{j} \left( t \right) + \delta _{j} r_{j} \left( t \right)} \right) - \left( {\varepsilon _{j}  + \zeta _{j}  + \lambda _{j} } \right)i_{j} \left( t \right) $$

3 $$  \dot{d}_{j} \left( t \right) = \varepsilon _{j} i_{j} \left( t \right) - \left( {\eta _{j}  + \rho _{j} } \right)d_{j} \left( t \right)  $$

4 $$  \dot{a}_{j} \left( t \right) = \zeta _{j} i_{j} \left( t \right) - \left( {\theta _{j}  + \mu _{j}  + \kappa _{j} } \right)a_{j} \left( t \right)$$

5 $$   \dot{r}_{j} \left( t \right) = \eta _{j} d_{j} \left( t \right) + \theta _{j} a_{j} \left( t \right) - \left( {\nu _{j}  + \xi _{j} } \right)r_{j} \left( t \right)   $$

6 $$  \dot{t}_{j} \left( t \right) = \mu _{j} a_{j} \left( t \right) + \nu _{j} r_{j} \left( t \right) - \left( {\sigma _{j}  + \tau _{j} } \right)t_{j} \left( t \right)  $$

7 $$  \begin{aligned}   \dot{H}\left( t \right) =  & \mathop \sum \limits_{{j = 1,2}} \left( {\lambda _{j} i_{j} \left( t \right) + \rho _{j} d_{j} \left( t \right) + \kappa _{j} a_{j} \left( t \right) + \xi _{j} r_{j} \left( t \right) + \sigma _{j} t_{j} \left( t \right)} \right) - \omega H\left( t \right) \\     & ~\mathop \sum \limits_{{j = 1,2}} \left( {\alpha _{j} i_{j} \left( t \right) + \beta _{j} d_{j} \left( t \right) + \gamma _{j} a_{j} \left( t \right) + \delta _{j} r_{j} \left( t \right)} \right) \\  \end{aligned}   $$

8 $$ \dot{E}\left( t \right) = \mathop \sum \limits_{{j = 1,2}} \tau _{j} t_{j} \left( t \right) $$

In the equations, the Latin letters represent the actual number of individuals in each stage of the pandemic at a certain time. *S*, susceptible; *I*_*j*_(*j* = 1, 2), total infected of the *j*th virus strain; and *R*, removed. The infected population can be further divided into 5 subgroups: *i*_*j*_, infected (asymptomatic infected, undetected); *d*_*j*_, diagnosed (asymptomatic infected, detected); *a*_*j*_, ailing (symptomatic infected, undetected); *r*_*j*_, recognized (symptomatic infected, detected); and *t*_*j*_, threatened (infected with life-threatening symptoms, detected). The stage *R* is further divided into two subgroups: *H* for the healed, and *E* for the extinct or deceased. Arrows from *S* to *I*_*j*_ only enter *i*_*j*_, while arrows from *I*_*j*_ to *R* are from all 5 subgroups of *I*_*j*_, and the dashed arrows from *H* to *I*_*j*_ represent the small chance of healed individuals becoming reinfected. The Greek letters represent the transition probability at each time step between different stages, which are regarded as positive constants in the whole simulation.

1. $\alpha _{j}\, \beta _{j}\, \gamma _{j}$, and $\delta _{j}$ (*j* = 1, 2) represent the infection rate caused by four different subgroups of infections at each time step. Generally, people tend to avoid contact with symptomatic infected or diagnosed individuals, so the value of $\gamma _{j}$ is slightly less than that of $\alpha _{j}$, while $\beta _{j}$ and $\delta _{j}$ caused by diagnosed patients are the smallest. In the simulation, Block policy can reduce all four infection rates simultaneously in equal proportion.
2. ${\varepsilon_j}$ and ${\theta_j}$ $\left( {j = 1,2} \right)$ are the diagnosis rates of the asymptomatic and symptomatic individuals. Compared with the asymptomatic patients, the symptomatic patients are more likely to go to the hospital for an evaluation, so the diagnosis rate will be higher for them than for asymptomatic patients. The Screen policy can change the diagnosis rate value. The two parameters were tuned based on a previous study [34], which showed that approximately half of the infected patients eventually developed symptoms (see Parameter tuning section at the end of Methods).
3. ${\zeta_j}$ and ${\eta_j}\left( {j = 1,2} \right)$ are the rates of asymptomatic patients developing clinically relevant symptoms. The present model assumes that these two rates are mainly affected by the severity of the virus strain; the more serious the virus strain is, the more likely it is to cause symptoms.
4. ${\mu_j}$ and ${\nu_j}$ $\left( {j = 1,2} \right)$ represent the occurrence rate of severe life-threatening symptoms in symptomatic infected patients. Good care, such as the utilization of Mobile Cabin Hospitals can effectively reduce the occurrence rate of severe symptoms. Different severities of the virus strains will affect these parameters as well.
5. ${\tau_j}$ $\left( {j = 1,2} \right)$ is the mortality rate of threatened patients. According to the existing data, the average mortality rate of the virus is set to 1% [35].
6. ${\lambda_j}$, ${\kappa_j}$, ${\xi_j}$,${\rho_j}$ and ${\sigma_j}$ $\left( {j = 1,2} \right)$ denote the cure rates for the five subgroups of the infected. The cure rates are affected by the severity of virus strains and patient symptoms. Given appropriate treatment or Cure policy, these values will be tuned proportionally.
7. $\omega$ is the reduction ratio for healed individuals to become infected compared to the susceptible population $S$. At present, the number of secondary infections is set to 0.01.

### Strain competition component of the coSIR model

The strain competition component is mainly based on the assumption that both virus strains can stimulate immune responses that are cross-reactive to each other. Thus, the two virus strains share the same susceptible population. As shown in the equations, each strain has a different $i,\;d,\;a,\;r,\;t$ while sharing the common $S,\;H$ and $E$.

In our simulation, both strains were influenced by the policies or control levels, while severity and transmissibility were changed for the emerging strain only. The severity and transmissibility are based on the ratio between the emerging strain and the original strain. When the ratio = 1, the two strains have the same severity or transmissibility. When the ratio > 1, the emerging strain has higher severity or transmissibility than the original strain, and when the ratio < 1, the emerging strain’s severity or transmissibility is lower. Throughout the pandemic, the overall infections (${\text{OI}}_{\text{j}}$) and overall mortality (${\text{OM}}_{\text{j}}$) of the $j$th virus can be calculated as

9 $$ \begin{aligned}   {\text{OI}}_{j}  =  & \int\limits_{0}^{{t_{{{\text{end}}}} }} {S\left( t \right)\left( {~\alpha _{j} i_{j} \left( t \right) + \beta _{j} d_{j} \left( t \right) + \gamma _{j} a_{j} \left( t \right) + \delta _{j} r_{j} \left( t \right)} \right)dt}  \\     &  + \int\limits_{0}^{{t_{{{\text{end}}}} }} {\omega H\left( t \right)\left( {~\alpha _{j} i_{j} \left( t \right) + \beta _{j} d_{j} \left( t \right) + \gamma _{j} a_{j} \left( t \right) + \delta _{j} r_{j} \left( t \right)} \right)dt}  \\  \end{aligned}  $$

10 $$ {\text{OM}}_{j}  = E_{j} \left( {t_{{{\text{end}}}} } \right) = \int\limits_{0}^{{t_{{{\text{end}}}} }} {\tau _{j} t_{j} \left( t \right)dt.}   $$

Considering that

11 $$ \begin{aligned} {{\dot H}_j}\left( t \right) = & {\lambda_j}{i_j}\left( t \right) + {\rho_j}{d_j}\left( {\text{t}} \right) + {\kappa_j}{a_j}\left( {\text{t}} \right) + {\xi_j}{r_j}\left( {\text{t}} \right) + {\sigma_j}{t_j}\left( {\text{t}} \right) \\ & - \omega {H_j}\left( t \right)\left( {\mathop \sum \limits_{k = 1,2} {\alpha_k}{i_k}\left( t \right) + {\beta_k}{d_k}\left( t \right) + {\gamma_k}{a_k}\left( t \right) + {\delta_k}{r_k}\left( t \right)\;} \right) \\ \end{aligned} $$

The formula (9) could be simplified into

12 $${\text{O}}{{\text{I}}_j} = {H_j}\left( {{t_{{\text{end}}}}} \right) + {\text{O}}{{\text{M}}_j} $$

The simulation assumes that when $t = {t_{{\text{end}}}}$ the epidemic is over, and the boundary conditions are ${I_j}\left( {t = 0} \right) = 0$ and ${I_j}\left( {t = {t_{{\text{end}}}}} \right) = 0$. The scores $\lg \left( {{\text{O}}{{\text{I}}_2}/{\text{O}}{{\text{I}}_1}} \right)$ or $\lg \left( {{\text{O}}{{\text{M}}_2}/{\text{O}}{{\text{M}}_1}} \right)$ can be used as indicators for the result of the competition between the strains. However, it is worth noting that the emerging strain may not reach the given initial threshold of starting competition when its transmissibility is too weak or when the policy inhibitions are too strong. In this case, the emerging strain cannot spread, resulting in ${\text{O}}{{\text{I}}_2} = 0$ or ${\text{O}}{{\text{M}}_2} = 0$. To avoid infinities caused by taking the logarithm of 0, the scores used to draw the three-dimensional images are tuned as

13 $${C_{{\text{infection}}}} = \lg \left( {\frac{{{\text{O}}{{\text{I}}_2} + {\varepsilon_0}}}{{{\text{O}}{{\text{I}}_1} + {\varepsilon_0}}}} \right)$$

14 $$ {C_{{\text{mortality}}}} = \lg \left( {\frac{{{\text{O}}{{\text{M}}_2} + {\varepsilon_0}}}{{{\text{O}}{{\text{M}}_1} + {\varepsilon_0}}}} \right),$$

where ${\varepsilon_0}$ is a small constant that won’t influence the scores when ${\text{O}}{{\text{I}}_2} \ne 0$ or ${\text{O}}{{\text{M}}_2} \ne 0$, and is set to ${\varepsilon_0} = {10^{ - 10}}$ in the simulations.

*Control levels* The four different control levels considered in the present model are based on the fitting results of the SARS-CoV-2 infection data in Italy [19] and modified according to the recent development of the pandemic. According to the restriction strength on the transmission speed of the virus, the four levels of virus control, from weak to strong, are named as Free Development, Weak Control, Medium Control and Strict Control. Free Development (${\alpha_j} = 0.5700,\;\;{\beta_j} = {\delta_j} = 0.1140,\;\;{\gamma_j} = 0.4560$) has no restrictions on virus transmission, which corresponds to the initial epidemic development in the Italian model. Weak Control (${\alpha_j} = 0.4218,\;\;{\beta_j} = {\delta_j} = 0.0570,\;\;{\gamma_j} = 0.2850$) corresponds to the basic social distance stage in the Italian model, where preliminary transmission control, such as maintaining social distance, hygiene and behavioural recommendations, is carried out, with ${\beta_j}$ and ${\delta_j}$ tuned. Moreover, under Weak Control, the symptomatic infected individuals will be self-isolated. Strict Control (${\alpha_j} = 0.2100,\;\;{\beta_j} = {\delta_j} = 0.0050,\;\;{\gamma_j} = 0.1100$) also implements population flow control, nucleic acid screening, the isolation of diagnosed patients and other medical measures, corresponding to the broader diagnosis campaign stage in the Italian model. The Medium Control (${\alpha_j} = 0.2700,\;\;{\beta_j} = {\delta_j} = 0.0232,\;\;{\gamma_j} = 0.1677$) level is also modelled to simulate virus transmission in various situations more comprehensively. Under this level, population control is implemented, but the isolation and screening efforts are not as strong as those under Strict Control. Specific parameter implications and adjustments in accordance with other studies have been explained in the epidemic dynamics of the coSIR model.

*Policies* Six common virus management policies included in our simulations are Block, Cure, Isolation, Screen, Isolation and Screen and Mobile Cabin Hospitals (Table S2). ‘Block’ reduces the infection rate of the two competing strains in equal proportions in the model, corresponding to social distancing, mask-wearing in public places and other population flow control policies. The parameters ${\alpha_j}$, ${\beta_j}$, ${\gamma_j}$ and ${\delta_j}$ are inversely proportional to the Block policy strength. ‘Cure’ means increasing the cure rate of COVID-19 patients, including improving the level of medical care and developing specific drugs. The parameters ${\lambda_j}$, ${\kappa_j}$, ${\xi_j}$, ${\rho_j}$ and ${\sigma_j}$ are proportional to the Cure policy strength. ‘Isolation’ means isolating the diagnosed patients, and the parameters ${\beta_j}$ and ${\delta_j}!$ are inversely proportional to Isolation policy strength. ‘Screen’ refers to conducting nucleic acid screening for a part of the population and can proportionally change the diagnosis rate (${\varepsilon_j}$ and ${\theta_j}$) of symptomatic and asymptomatic patients equally. ‘Isolation & Screen’ is a combination of the Isolation and the Screen policies of equal rate. ‘Mobile Cabin Hospitals’ is a combination of using isolation and reducing the occurrence of life-threatening symptoms. In the simulation, the life-threatening symptom occurrence rate and the cure rate of diagnosed individuals change inversely with the policy strength, while the isolation rate is fixed at a very small value (${\beta_j} = {\delta_j} = 0.001$).

*Virus variants* The emerging virus strain differs from the original strain in two aspects: severity and transmissibility (Table S2). Severity affects the symptomatic rates (${\zeta_j}$ and ${\eta_j}$), severe life-threatening symptom rates (${\mu_j}$ and ${\nu_j}$), mortality (${\tau_j}$) and healing rates (${\lambda_j}$, ${\kappa_j}$, ${\xi_j}$, ${\rho_j}$ and ${\sigma_j}$). Furthermore, the symptomatic, life-threatening and mortality rates are positively proportional to the severity ratio, while the healing rates are negatively correlated with the severity ratio (formulas 15, 16). Transmissibility proportionally affects the infection rate (${\alpha_j}$, ${\beta_j}$, ${\gamma_j}$ and ${\delta_j}$).

15 $$\left( {{\zeta_j},{\eta_j},{\mu_j},\;{\nu_j},\;{\tau_j}} \right) \leftarrow \left( {{\zeta_j},{\eta_j},{\mu_j},\;{\nu_j},\;{\tau_j}} \right) \cdot {\text{rati}}{{\text{o}}_{{\text{severity}}}} $$

16 $$\left( {{\lambda_j},{\kappa_j},\;{\xi_j},\;{\rho_j},\;{\sigma_j}} \right) \leftarrow \left( {{\lambda_j},{\kappa_j},\;{\xi_j},\;{\rho_j},\;{\sigma_j}} \right) \cdot \left( {1.1 - 0.1 \cdot {\text{rati}}{{\text{o}}_{{\text{severity}}}}} \right) $$

*Initial status* The time point when the emerging strain enters the competition, i.e., the initial infection ratio between the original strain and the emerging strain, matters in the whole competitive dynamics. To determine the initial infection ratio, we first simulated the dynamics of each of the two strains under the Free Development control level separately to reach a certain number of infections, such as 10,000 existing infections for the original strain and 100 for the emerging strain. Next, all the variables of the two strains were measured and then combined to obtain a competitive system. Different simulations showed that the initial infection ratio between the original strain and the emerging strain had no significant impact on the results of strain competition in most cases (Fig. S1). Therefore, the ratio 10,000:100 is used in all the simulations for simplification.

*Parameter tuning* Most of the parameters were adopted from the SIDARTHE model, with some changes [19]. The potential of developing symptoms, being cured, and being diagnosed for an asymptomatic infected individual at every time step is ${\zeta_j}$, ${\lambda_j}$ and ${\varepsilon_j}$, and the overall symptomatic rate is $\frac{{\zeta_j}}{{{\zeta_j} + {\lambda_j} + {\varepsilon_j}}}$. The diagnosis rate (${\varepsilon_j}$) was set to 0.1026 to ensure an overall symptomatic rate of approximately 50%. With this change, the undetected individuals comprised approximately half of the total infections under Free Development before the infections peak, which is consistent with the results from Iceland [34]. By changing the mortality rate at each time step from 0.001 to 0.0001, the overall mortality in the whole population was reduced to ~ 0.3% under Free Development. In addition, we also changed the infection rate caused by diagnosed individuals (${\beta_j},\;{\delta_j}$) from 0.0057 to 0.057 under Weak Control to distinguish it from the Strict Control when the rate was set to 0.050. In fact, the conclusions here are robust and are not significantly influenced by the changes in the parameters (Fig. S6).

### Computation of ${R_0}$

Equations (1)–(8) can be rewritten into the form of a positive bilinear system

17 $$ {\dot x_j}\left( t \right) = {F_j}{x_j}\left( t \right) + b{u_j}\left( t \right) $$

18 $$ \dot S\left( t \right) = \mathop \sum \limits_{j = 1,2} {y_{S_j}}\left( t \right) $$

19 $$ \dot H\left( t \right) = \mathop \sum \limits_{j = 1,2} {y_{H_j}}\left( t \right) $$

20 $$ \dot E\left( t \right) = \mathop \sum \limits_{j = 1,2} {y_{E_j}}\left( t \right) $$

21 $${u_j}\left( {\text{t}} \right) = \left( {\omega H\left( {\text{t}} \right) + S\left( {\text{t}} \right)} \right) \cdot {c_j}^T{x_j}\left( {\text{t}} \right) $$

22 $$ {y_{S_j}}\left( t \right) = - S\left( t \right) \cdot {c_j}^T{x_j}\left( t \right)$$

23 $$ {y_{H_j}}\left( t \right) = {f_j}^T{x_j}\left( t \right) - \;\omega H\left( t \right) \cdot {c_j}^T{x_j}\left( t \right) $$

24 $$ {y_{E_j}}\left( t \right) = {d_j}^T{x_j}\left( t \right), $$

where

$$ {F_j} = \left[ {\begin{array}{*{20}{c}} { - {r_{j1}}}&0&0&0&0 \\ {\varepsilon_j}&{ - {r_{j2}}}&0&0&0 \\ {\zeta_j}&0&{ - {r_{j3}}}&0&0 \\ 0&{\eta_j}&{\theta_j}&{ - {r_{j4}}}&0 \\ 0&0&{\mu_j}&{\nu_j}&{ - {r_{j5}}} \end{array}} \right], $$

$$ \begin{aligned} {x_j}\left( t \right) & = {\left[ {\begin{array}{*{20}{c}} {{i_j}\left( t \right)}&{{d_j}\left( t \right)}&{{a_j}\left( t \right)}&{{r_j}\left( t \right)}&{{t_j}\left( t \right)} \end{array}} \right]^T}, \\ {c_j} & = {\left[ {\begin{array}{*{20}{c}} {\alpha_j}&{\beta_j}&{\gamma_j}&{\delta_j}&0 \end{array}} \right]^T}, \\ {d_j} & = {\left[ {\begin{array}{*{20}{c}} 0&0&0&0&{\tau_j} \end{array}} \right]^T}, \\ {f_j} & = {\left[ {\begin{array}{*{20}{c}} {\lambda_j}&{\rho_j}&{\kappa_j}&{\xi_j}&{\sigma_j} \end{array}} \right]^T}, \\ b & = {\left[ {\begin{array}{*{20}{c}} 1&0&0&0&0 \end{array}} \right]^T}, \\ {r_{j1}} & = {\varepsilon_j} + {\zeta_j} + {\lambda_j}, \\ {r_{j2}} & = {\eta_j} + {\rho_j}, \\ {r_{j3}} & = {\theta_j} + {\mu_j} + {\kappa_j}, \\ {r_{j4}} & = {\nu_j} + {\xi_j}\;{\text{and}}\; \\ {r_{j5}} & = {\sigma_j} + {\tau_j} \\ \end{aligned}  $$

At each time step, we have

25 $$ \mathop \sum \limits_{j = 1,2} \left[ {\begin{array}{*{20}{c}} {1,}&{1,}&{1,}&{1,}&1 \end{array}} \right] \cdot {x_j}\left( t \right) + S\left( t \right) + H\left( t \right) + E\left( t \right) = 1$$

Measuring the stability of the disease-free equilibrium can helps inferring the basic reproduction number ${R_0}$. Under the disease-free equilibrium, we have $\left( {t_\infty } \right) = \bar S,\;\;H\left( {t_\infty } \right) = \bar H,\;\;E\left( {t_\infty } \right) = \bar E$ and $${x_j}\left( {t_\infty } \right) = {\left[ {\begin{array}{*{20}{c}} {0,}&{0,}&{0,}&{0,}&0 \end{array}} \right]^T}$$. The Jacobian matrix of the linearized system around the equilibrium is

26 $$ J = \left[ {\begin{array}{*{20}{c}} 0&{ - \bar S{c_1}^T}&{ - \bar S{c_2}^T}&0&0 \\ 0&{J_1}&0&0&0 \\ 0&0&{J_2}&0&0 \\ 0&{{f_1}^T - \omega \bar H{c_1}^T}&{{f_2}^T - \omega \bar H{c_2}^T}&0&0 \\ 0&{{d_1}^T}&{{d_2}^T}&0&0 \end{array}} \right], $$

where ${J_i} = \left( {\omega \bar H + \bar S} \right){c_i}^Tb + {F_i}$, whose detailed form [19] is

$$ J_{j}  = \left[ {\begin{array}{*{20}c}    {\alpha _{i} \left( {\omega \bar{H} + \bar{S}} \right) - r_{{j1}} } & {\beta _{i} \left( {\omega \bar{H} + \bar{S}} \right)} & {\gamma _{i} \left( {\omega \bar{H} + \bar{S}} \right)} & {\delta _{i} \left( {\omega \bar{H} + \bar{S}} \right)} & 0  \\    {\varepsilon _{i} } & { - r_{{j2}} } & 0 & 0 & 0  \\    {\zeta _{i} } & 0 & { - r_{{j3}} } & 0 & 0  \\    0 & {\eta _{i} } & {\theta _{i} } & { - r_{{j4}} } & 0  \\    0 & 0 & {\mu _{i} } & {\nu _{i} } & { - r_{{j5}} }  \\   \end{array} } \right] $$

Note that the Jacobian matrix $J$ is partitioned, and thus has three null eigenvalues and ten eigenvalues derived from the eigenvalues of ${J_1}$ and ${J_2}$. The eigenvalues of matrix $J$ satisfy the function

27 $$p\left( s \right) = {p_1}\left( s \right){p_2}\left( s \right) = ({D_1}\left( s \right) + \left( {\omega \bar H + \bar S} \right){N_1}\left( s \right))\left( {{D_2}\left( s \right) + \left( {\omega \bar H + \bar S} \right){N_2}\left( s \right)} \right)$$

where

$$ {D_j}\left( s \right) = \left( {s + {r_{j1}}} \right)\left( {s + {r_{j2}}} \right)\left( {s + {r_{j3}}} \right)\left( {s + {r_{j4}}} \right)\left( {s + {r_{j5}}} \right) $$

$$ \begin{aligned}   N_{j} \left( s \right) =  & \left( {s + r_{{j5}} } \right) \\     & \left( \begin{gathered}   \alpha _{j} \left( {s + r_{{j2}} } \right)\left( {s + r_{{j3}} } \right)\left( {s + r_{{j4}} } \right) + \beta _{j} \varepsilon _{j} \left( {s + r_{{j3}} } \right)\left( {s + r_{{j4}} } \right) \hfill \\    + \gamma _{j} \zeta _{j} \left( {s + r_{{j2}} } \right)\left( {s + r_{{j4}} } \right) + \delta _{j} \eta _{j} \varepsilon _{j} \left( {s + r_{{j3}} } \right) + \delta _{j} \zeta _{j} \theta _{j} \left( {s + r_{{j2}} } \right) \hfill \\  \end{gathered}  \right) \\  \end{aligned}  $$

As has been calculated in the SIDARTHE model [19], the polynomial ${p_j}$ is Hurwitz (all roots in the left-hand plane) iff

$$ \omega \bar H + \bar S < {\left( {\frac{{\alpha_j}}{{{r_{j1}}}} + \frac{{{\beta_j}{\varepsilon_j}}}{{{r_{j1}}{r_{j2}}}} + \frac{{{\gamma_j}{\zeta_j}}}{{{r_{j1}}{r_{j3}}}} + \frac{{{\delta_j}{\eta_j}{\varepsilon_j}}}{{{r_{j1}}{r_{j2}}{r_{j4}}}} + \frac{{{\delta_j}{\zeta_j}{\theta_j}}}{{{r_{j1}}{r_{j3}}{r_{j4}}}}} \right)^{ - 1}}$$

Then, the reproduction number of each strain is

$${R_{0j}} = \frac{1}{\omega \bar H + \bar S}\left( {\frac{{\alpha_j}}{{{r_{j1}}}} + \frac{{{\beta_j}{\varepsilon_j}}}{{{r_{j1}}{r_{j2}}}} + \frac{{{\gamma_j}{\zeta_j}}}{{{r_{j1}}{r_{j3}}}} + \frac{{{\delta_j}{\eta_j}{\varepsilon_j}}}{{{r_{j1}}{r_{j2}}{r_{j4}}}} + \frac{{{\delta_j}{\zeta_j}{\theta_j}}}{{{r_{j1}}{r_{j3}}{r_{j4}}}}} \right), $$

and the threshold condition ${R_0}$ for the stability of the disease-free equilibrium is

$${R_0} = {\text{max\;}}\left( {{R_{01}},{R_{02}}} \right)$$

If ${R_0} < 1$, then ${R_{01}} < 1$ and ${R_{02}} < 1$, namely, the polynomial ${p_1},{p_2}$ is Hurwitz so that $p\left( s \right)$ is Hurwitz and the disease-free equilibrium is locally asymptotically stable. Similarly, the disease-free equilibrium is unstable if ${R_0} \geqslant 1$. That is, the stability at the disease-free equilibrium is determined by the strain infecting more individuals.

The ${R_0}$ can also be calculated as

$$ {R_0} = \frac{{{I_1}{R_{01}} + {I_2}{R_{02}}}}{{{I_1} + {I_2}}},{\text{\;}} $$

where ${I_1}$ and ${I_2}$ are the numbers of infections of two strains respectively, which satisfies $0 < {I_1} < 1{\text{\;and\;}}0 < {I_2} < 1$. If the emerging strain infects a negligible number of individuals compared to the original strain, i.e., ${I_1} \gg {I_2}$, then ${R_0} \approx {R_{01}}$; if ${I_1} \ll {I_2}$, ${R_0} \approx {R_{02}}$; if the two strains cause similar number of infections, then ${R_0} \approx {R_{01}} \approx {R_{02}}$.

## Supplementary Information

Below is the link to the electronic supplementary material.

Supplementary file1 (DOCX 4954 kb)

## Data Availability

Data sharing not applicable to this article as no datasets were generated or analysed during the current study.
