# Supplementary material for: The coSIR model predicts effective strategies to limit the spread of SARS-CoV-2 variants with low severity and high transmissibility
Source: Nonlinear Dyn. 2021 Jul 23;105(3):2757–73. doi: 10.1007/s11071-021-06705-8 (PMC8300993; doi:10.1007/s11071-021-06705-8)
Supplement: Supplementary file 1 — Supplementary file1 (DOCX 4954 kb) [file 11071_2021_6705_MOESM1_ESM.docx]

**SUPPLEMENTARY MATERIALS**

**
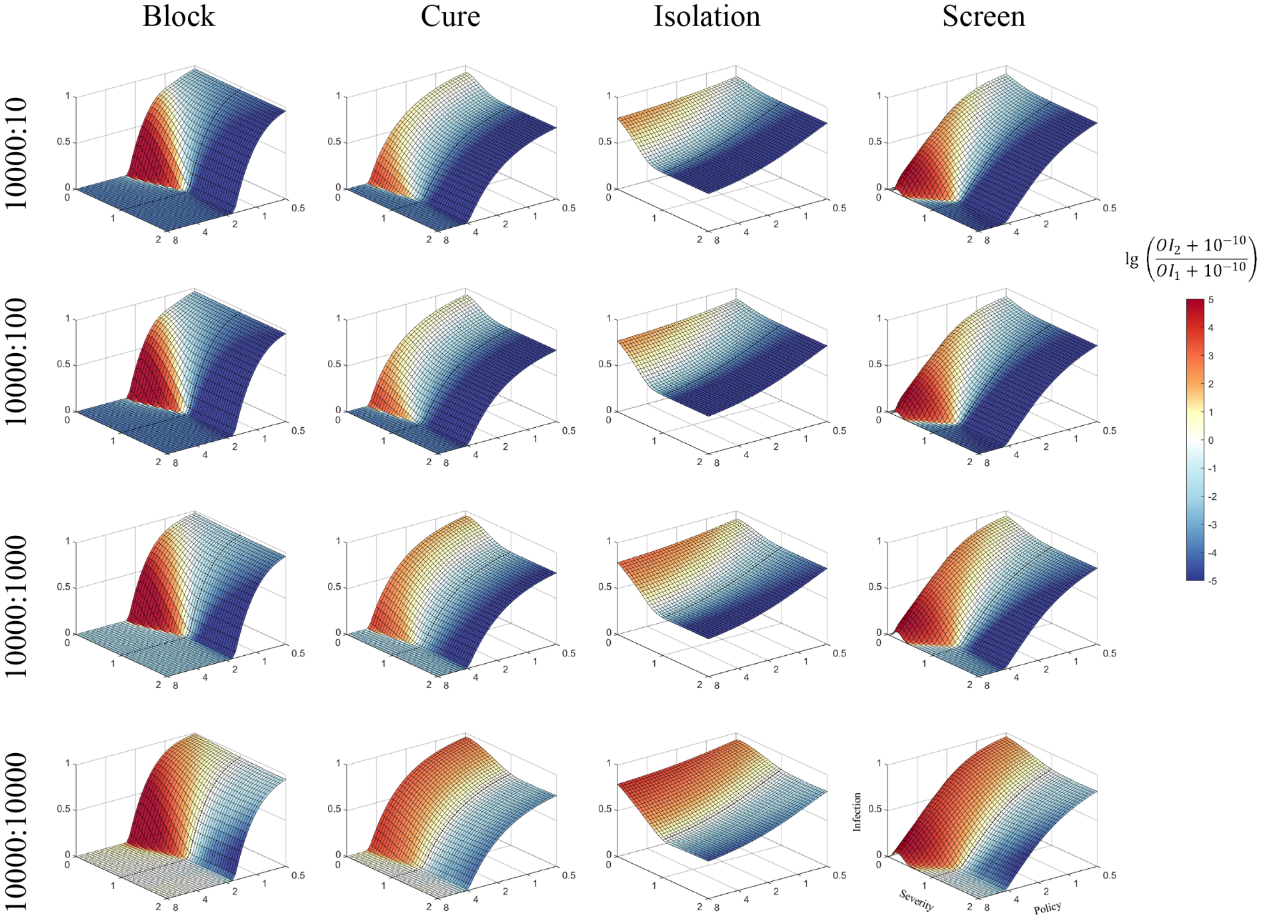
Fig. S1 | Competition under different initial infection ratios suggesting the robustness of the simulation results.** Under different policies and initial infection ratios between the two strains, all competition results share the same pattern, indicating that the impact of the initial infection ratio on the competition dynamics is not significant. In the following analyses, we set the initial infection ratio of the original strain to the new strain at 10000:100 for simplification.

**
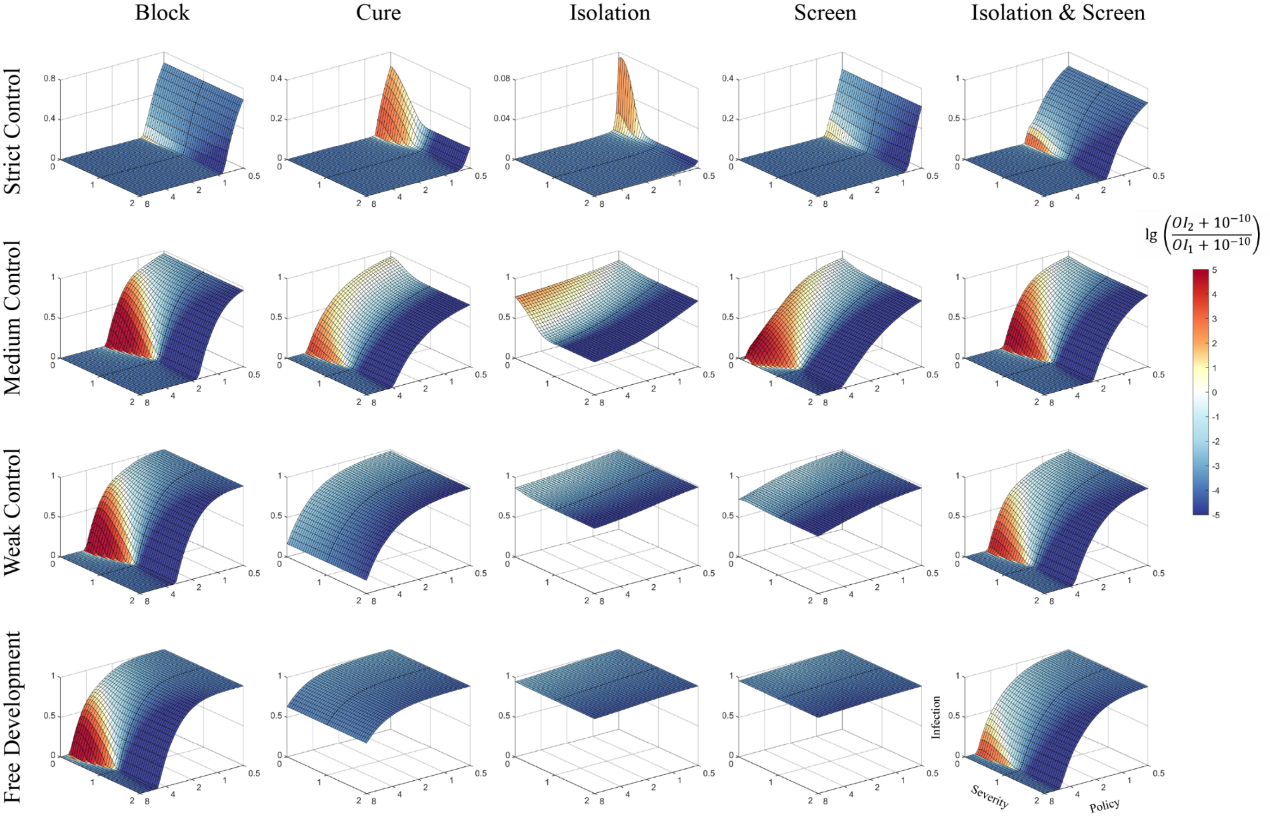
Fig. S2 | Competition between virus strains with different symptom severities.** The simulation results under different policies (Block, Cure, Isolation, Screen, Isolation & Screen) and different virus control levels (Strict Control, Medium Control, Weak Control, Free Development). The x-axis represents the severity of the emerging strain relative to that of the original strain, which is set to 1. A value < 1 means that the severity of the emerging strain is lower than that of the original strain and vice versa. The y-axis represents policy strengths, which is the factor used to multiply (or divide) the specific parameters related to the four respective policies in the coSIR model (for policy-related parameters, see Methods and Table S2). The z-axis represents the overall infection rate among the whole population. The colour bar represents the log ratio of the number of overall infected individuals ($OI$) of the emerging strain to that of the original strain. A positive number means that the emerging strain outcompetes the original strain in terms of the number of infections.

**
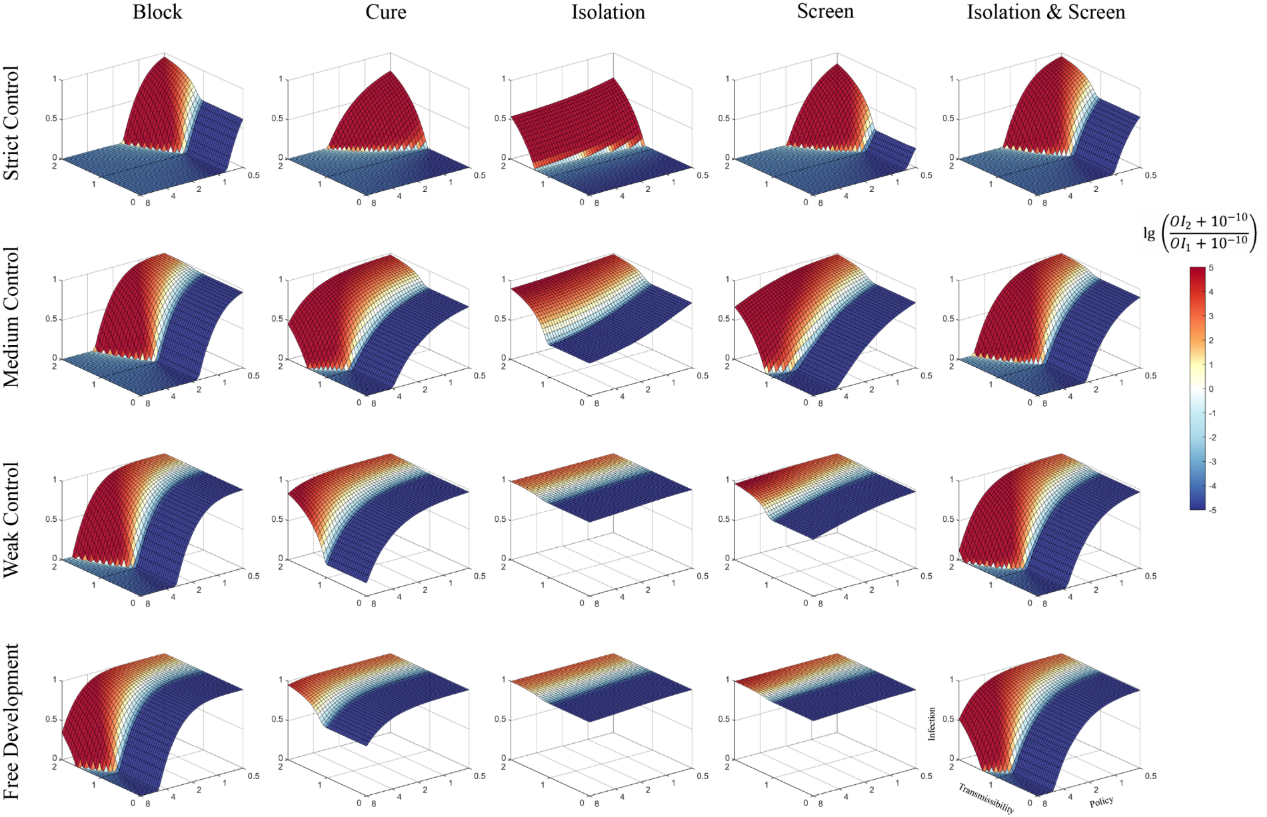
Fig. S3 | Competition between virus strains with different transmissibilities.** The predicted infection rate of the new strain is always positively correlated with its transmissibility under various policies and control levels.

**
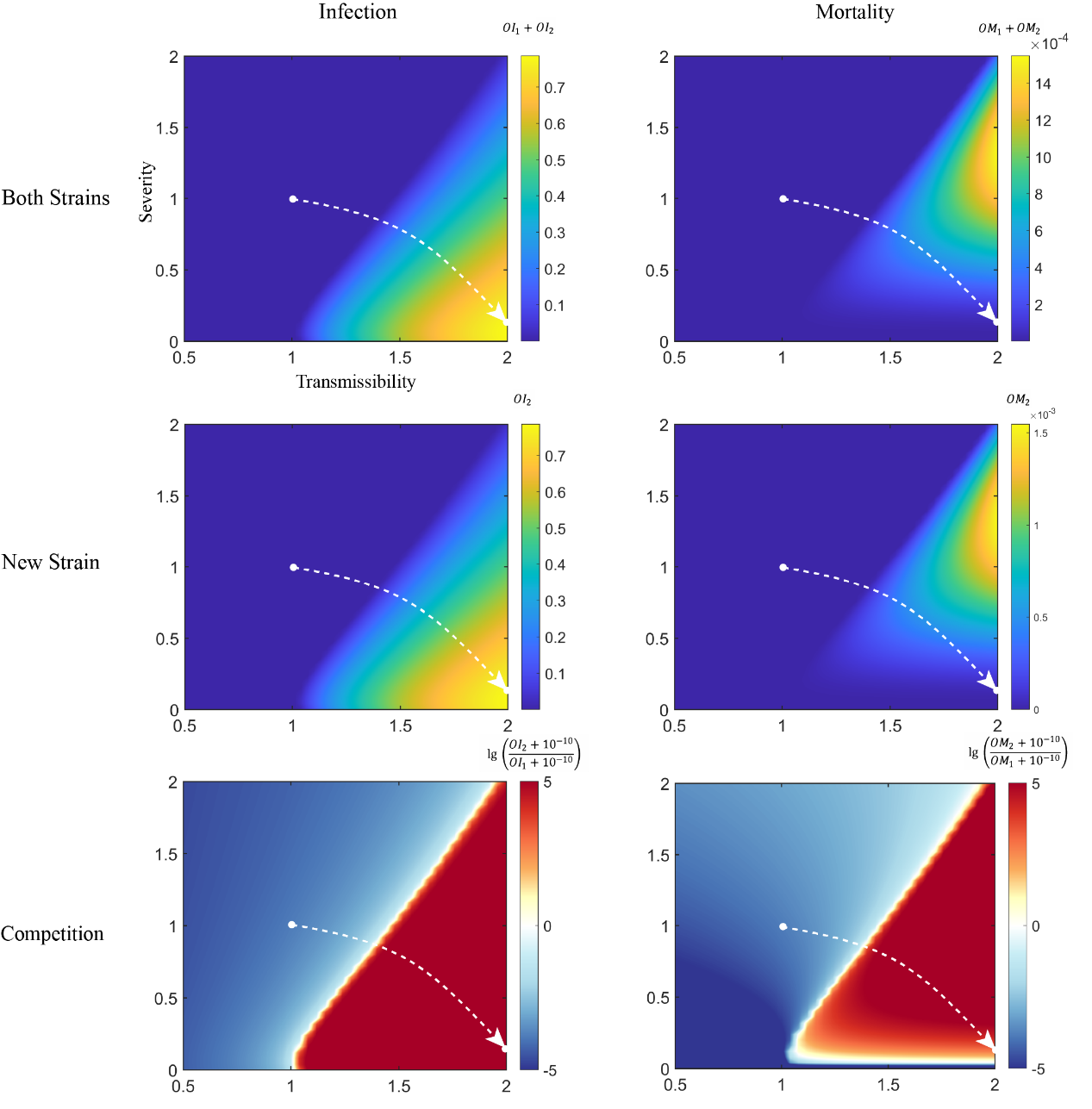
Fig. S4 | Infection and mortality of virus strains with different transmissibility and severity.** The top panels are the summed results of both the original strain and the emerging strain, and the middle panels are the results of the emerging strain alone. Bottom panels show the competition results of the two strains in terms of the relative number of infections (left) and number of deaths (right). When the emerging strain outcompetes the original strain, the infection/mortality of both strains are at a high level and vice versa.

**
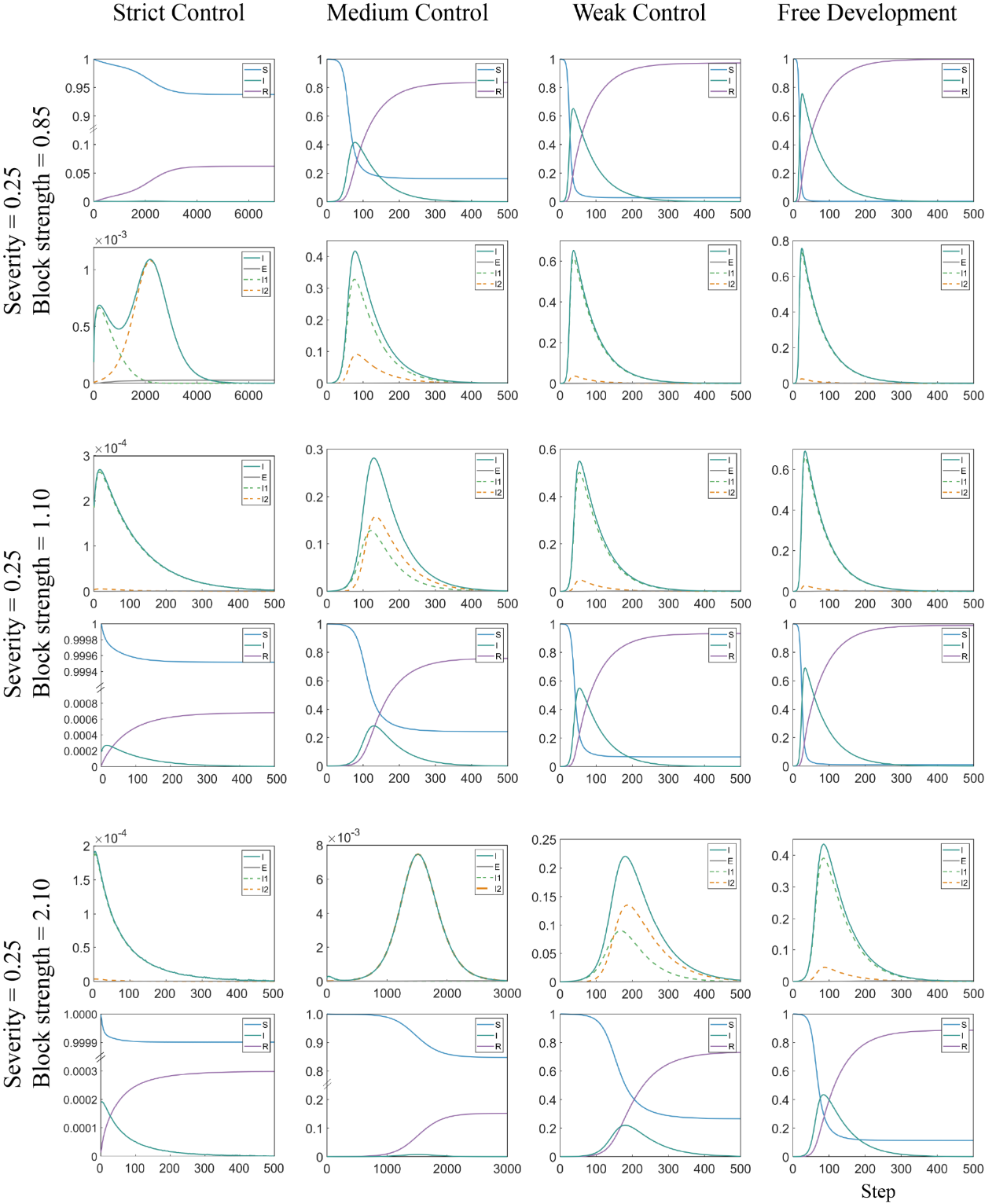
**

**Fig. S5 | SIR curves of several examples under different Block policy strengths and control levels.** For the emerging strain with the same severity given specific policy strengths, different control levels produced distinct competition dynamics.

**
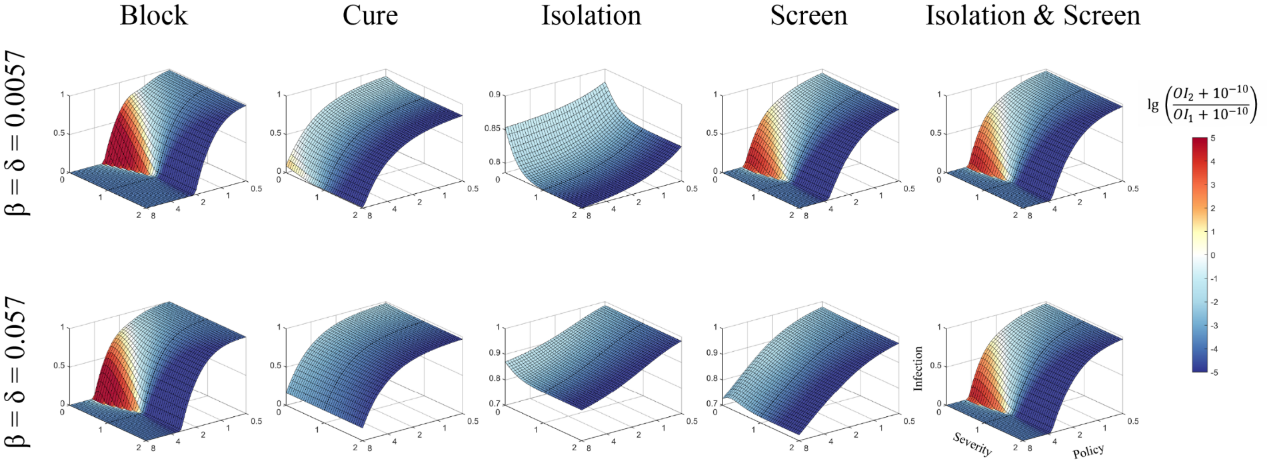
Fig. S6 | Comparison of competition results given two different probabilities of a susceptible individual being infected after being in contact with a diagnosed individual under Weak Control.** The upper panels show the results under the probabilities set in the SIDARTHE model, while the bottom panels show the results under the probabilities in the coSIR model. Similar patterns were observed except that in the upper panels, the emerging strains were even more advantageous. We used the parameters in the bottom panels in the coSIR model because the parameters of the SIDARTHE model under Weak Control are too close to those under Strict Control (Table S1).

|  | Free Development | Weak Control | Medium Control | Strict Control |
| --- | --- | --- | --- | --- |
| α | 0.5700 | 0.4218 | 0.2700 | 0.2100 |
| β | 0.1140 | 0.0570 | 0.0232 | 0.0050 |
| γ | 0.4560 | 0.2850 | 0.1677 | 0.1100 |
| δ | 0.1140 | 0.0570 | 0.0232 | 0.0050 |
| ε | 0.1026 | 0.1026 | 0.1026 | 0.2000 |
| θ | 0.3705 | 0.3705 | 0.3705 | 0.3705 |
| ζ | 0.1254 | 0.1254 | 0.1254 | 0.0250 |
| η | 0.1254 | 0.1254 | 0.1254 | 0.0250 |
| μ | 0.0171 | 0.0171 | 0.0171 | 0.0080 |
| ν | 0.0274 | 0.0274 | 0.0274 | 0.0150 |
| τ | 0.0001 | 0.0001 | 0.0001 | 0.0001 |
| λ | 0.0342 | 0.0342 | 0.0342 | 0.0800 |
| ρ | 0.0342 | 0.0342 | 0.0342 | 0.0200 |
| κ | 0.0171 | 0.0171 | 0.0171 | 0.0200 |
| ξ | 0.0171 | 0.0171 | 0.0171 | 0.0200 |
| σ | 0.0171 | 0.0171 | 0.0171 | 0.0100 |
| ω | 0.0100 | 0.0100 | 0.0100 | 0.0100 |

**Table S1 | Default parameters for each control level**

**Table S2 | Directions of parameter adjustment under increased policy strength and with an emerging strain of higher transmissibility and severity**

|  | Policy* | | | | | | Strain characteristics** | |
| --- | --- | --- | --- | --- | --- | --- | --- | --- |
|  | Block | Cure | Isolation | Screen | Isolation & Screen | Cabin Hospital | Transmissibility | Severity |
| *α* | ↓ | - | - | - | - | - | ↑ | - |
| *β* | ↓ | - | ↓ | - | ↓ | ↓ | ↑ | - |
| *γ* | ↓ | - | - | - | - | - | ↑ | - |
| *δ* | ↓ | - | ↓ | - | ↓ | ↓ | ↑ | - |
| *ε* | - | - | - | ↑ | ↑ | - | - | - |
| *θ* | - | - | - | ↑ | ↑ | - | - | - |
| *ζ* | - | - | - | - | - | - | - | ↑ |
| *η* | - | - | - | - | - | - | - | ↑ |
| *μ* | - | - | - | - | - | ↓ | - | ↑ |
| *ν* | - | - | - | - | - | ↓ | - | ↑ |
| *τ* | - | - | - | - | - | - | - | ↑ |
| *λ* | - | ↑ | - | - | - | - | - | ↓ |
| *ρ* | - | ↑ | - | - | - | - | - | ↓ |
| *κ* | - | ↑ | - | - | - | - | - | ↓ |
| *ξ* | - | ↑ | - | - | - | - | - | ↓ |
| *σ* | - | ↑ | - | - | - | - | - | ↓ |
| *ω* | - | - | - | - | - | - | - | - |

* both strains are affected (upward arrows for multiplication by the default policy strength, downward arrows for division)

** only for the emerging strain (upward arrows for multiplication by the *ratio*, downward arrows for multiplication by $\left( 1.1-0.1\cdot ratio \right)$)
